# Morquio A syndrome and effect of enzyme replacement therapy in different age groups of Turkish patients: a case series

**DOI:** 10.1186/s13023-021-01761-0

**Published:** 2021-03-22

**Authors:** Sebile Kılavuz, Sibel Basaran, Deniz Kor, Fatma Derya Bulut, Sevcan Erdem, Hüseyin Tuğsan Ballı, Muhammed Dağkıran, Atil Bisgin, Halise Neslihan Önenli Mungan

**Affiliations:** 1 Division of Pediatric Metabolism and Nutrition, Department of Pediatrics, Faculty of Medicine, Çukurova University,, Adana, Turkey; 2 Department of Physical Medicine and Rehabilitation, Faculty of Medicine, Çukurova University, Adana, Turkey; 3 Division of Pediatric Cardiology, Department of Pediatrics, Faculty of Medicine, Çukurova University, Adana, Turkey; 4 Department of Radiology, Faculty of Medicine, Çukurova University, Adana, Turkey; 5 Department of Ear, Nose and Throat Diseases, Faculty of Medicine, Çukurova University, Adana, Turkey; 6 Medical Genetics Department of Medical Faculty, Cukurova University AGENTEM (Adana Genetic Diseases Diagnosis and Treatment Center), Adana, Turkey; 7 Division of Pediatric Metabolism and Nutrition, Department of Pediatrics, Faculty of Medicine, Çukurova University, 01130 Sarıçam, Adana, Turkey

**Keywords:** Elosulfase alfa, Enzyme replacement therapy, Mucopolysaccharidosis IVA, Morquio A syndrome

## Abstract

**Background:**

This case series includes longitudinal clinical data of ten patients with Morquio A syndrome from south and southeastern parts of Turkey, which were retrospectively collected from medical records. All patients received enzyme replacement therapy (ERT). Clinical data collected included physical appearance, anthropometric data, neurological and psychological examinations, cardiovascular evaluation, pulmonary function tests, eye and ear-nose-throat examinations, endurance in the 6-min walk test and/or 3-min stair climb test, joint range of motion, and skeletal investigations (X-rays, bone mineral density).

**Results:**

At the time of ERT initiation, two patients were infants (1.8 and 2.1 years), five were children (3.4–7.1 years), and three were adults (16.5–39.5 years). Patients had up to 4 years follow-up. Most patients had classical Morquio A, based on genotypic and phenotypic data. Endurance was considerably reduced in all patients, but remained relatively stable or increased over time in most cases after treatment initiation. Length/height fell below normal growth curves, except in the two infants who started ERT at ≤ 2.1 years of age. All patients had skeletal and/or joint abnormalities when ERT was started. Follow-up data did not suggest improvements in skeletal abnormalities, except in one of the younger infants. Nine patients had corneal clouding, which resolved after treatment initiation in the two infants, but not in the other patients. Hepatomegaly was reported in seven patients and resolved with treatment in five of them. Other frequent findings at treatment initiation were coarse facial features (N = 9), hearing loss (N = 6), and cardiac abnormalities (N = 6). Cardiac disease deteriorated over time in three patients, but did not progress in the others.

**Conclusions:**

Overall, this case series with Morquio A patients confirms clinical trial data showing long-term stabilization of endurance after treatment initiation across ages and suggest that very early initiation of ERT optimizes growth outcomes.

**Supplementary Information:**

The online version contains supplementary material available at 10.1186/s13023-021-01761-0.

## Background

Morquio A syndrome, also called mucopolysaccharidosis (MPS) IVA, is an ultra-rare autosomal recessive lysosomal storage disorder caused by deficient activity of the enzyme *N*-acetylgalactosamine-6-sulfatase (GALNS; EC 3.1.6.4), involved in the catabolism of the glycosaminoglycans (GAGs) keratan sulfate (KS) and chondroitin-6-sulfate [1]. The resulting accumulation of these GAGs in tissues and organs causes an array of multi-systemic and progressively worsening clinical manifestations [2].

Morquio A is typically characterized by skeletal dysplasia (dysostosis multiplex, spinal cord compression), short trunk dwarfism, joint abnormalities, impaired respiratory function, cardiac abnormalities, impaired vision, hearing loss, hepatomegaly, and a reduced lifespan [3–6]. Clinical manifestations and progression rates differ considerably between patients. Onset of disease symptoms can vary from prior to 1 year of age in severely affected (classical) patients to the second decade of life in less severely affected (non-classical) patients [1].

Therapeutic options for Morquio A syndrome include palliative symptom management and systemic therapies [7]. First-line therapy is elosulfase alfa, an enzyme replacement therapy (ERT), provided weekly (at 2 mg/kg) via intravenous infusions [8]. This therapy was developed to help correct the enzyme deficiency and restore cell function of Morquio A patients [9]. The pivotal phase 3 clinical trial of elosulfase alfa (MOR-004), including 176 patients ≥ 5 years of age, and its long-term extension (MOR-005) showed a statistically significant improvement in the distance walked in the 6-min walk test (6MWT), a rapid and sustained reduction in urine KS (uKS), and sustained numerical improvements in pulmonary function measures and activities of daily living after 120 weeks of treatment [8, 10–12]. Elosulfase alfa was generally well tolerated, with most drug-related adverse events being mild or moderate infusion-associated reactions [8, 10].

In Turkey, elosulfase alfa has been reimbursed since May 7, 2015 for patients > 60 months of age diagnosed with Morquio A by enzyme or mutation analysis, who can walk independently. On September 23, 2017, patients < 60 months of age and diagnosed by enzyme or mutation analysis also became eligible for reimbursement. To date, the Turkish Morquio A population and the impact of ERT on these patients have not been well defined.

## Methods

### Aims

The aims of reporting these cases were to better characterize the phenotypic and genotypic features of a small group of Turkish Morquio A patients, and to describe the clinical outcomes of these patients after initiating ERT at different ages in real-life clinical practice.

### Study design and participants

Clinical data of ten patients with a confirmed diagnosis of Morquio A syndrome who were followed up at the Çukurova University Balcalı Hospital in Adana, Turkey were retrospectively collected by a local clinical research officer from the patients’ medical records after ethical committee approval. Patients were selected as representatives of different age groups; and availability of clinical data was the second most important selection criterion. Clinical data that were collected included physical appearance, anthropometric data, neurological and mental/psychological examinations (Denver Developmental Screening Test II [up to age 6 years], Stanford-Binet test [children ≥ 2 years], Wechsler Intelligence Scale for Children Revised [WISC-R, 6–16 years], Wechsler Adult Intelligence Scale [WAIS, > 16 years]), cardiovascular evaluation, pulmonary function tests, eye and ear-nose-throat (ENT) examinations, endurance in the 6MWT and/or 3-min stair climb test (3MSCT) depending on age, joint range of motion, and skeletal investigations (X-rays, bone mineral density). All patients (and/or their parents) provided informed consent to publish clinical data of this case series.

### Statistical analysis

All data are presented descriptively and compared with normative data, where relevant and available. Z-scores (indicating how many standard deviation [SDs] a value deviates from the mean) were calculated for anthropometric data (length/height and weight).

## Results

### Demographics and patient characteristics

The case series included two infants (cases 1 and 2) aged 1.8 and 2.1 years, five children (cases 3–7) aged 3.4 to 7.1 years, and three adults (cases 8–10) aged 16.5 to 39.5 years at initiation of ERT (Table1). Consanguinity data showed that in all cases the patients’ parents were either first degree cousins (cases 1, 2, 4, 7, and 9), second degree cousins (cases 3, 5, 6 and 8) or distant relatives (case 10). Five patients (cases 1, 2, 3, 6, and 9) had a sibling with Morquio A syndrome.

**Table 1:** Patient demographics and baseline characteristics

Patient	Sex	Consanguinity of parents	Age at first symptoms (years)	Age at diagnosis (years)	Age at initiation of ERT (years)	Duration of ERT (years)	Current age (years)	Enzyme level nmol/mg/17 h (45–240)^a^	Mutation
1	M	Yes	1	0.04 (2 weeks)^b^	1.8	2.5	4.2	13	Homozygous: c.535 C > T (p. P179S)
2	M	Yes	1.1	1.5	2.1	4.2	6.3	0	Homozygous: c.421 T > A (p.V141R)
3	F	Yes	2	3.2	3.4	2.2	5.6	12	Homozygous: c.107 T > G (p.L36P)
4	F	Yes	2.5	3.7	4.0	2.9	6.9	0.1	Homozygous: c.268C > T (p.R90W)
5	F	Yes	2.8	4.9	5.2	2.8	8.0	0.1	Homozygous: c.922 T > C (p.C308R)
6	M	Yes	1.9	3.8	5.2^c^	3.0	8.2	18.8	Homozygous: c.655A > T (p.R219X)
7	F	Yes	1.5	5.4	7.1	4.6	11.7	13	Homozygous: c.421 T > A (p.W141R)
8	F	Yes	3	16.1	16.5	4.4	20.9	0	Homozygous: c.922 T > C (p.C308R)
9	M	Yes	7	15.6	16.6	4.5^d^	21.1	26	Homozygous: c.1417C > T (p.Q473X)
10	F	Yes	0.8	5.9	39.5	4.7	44.2	0	Homozygous: c.1348G > A (p.E450K)

^a^Determined before initiation of ERT; ^b^Treatment of case 1 started late because the family could not be reached; ^c^Case 6 was diagnosed in a different center, and his treatment was initially delayed due to hypersensitivity reactions; ^d^Case 9 was not compliant to the weekly treatment; he discontinued treatment many times; ERT: enzyme replacement therapy; F: female; M: male; NA: not available

Data were collected between August 2008 and July 2020. The number of visits at which data were collected ranged from seven (subject 4) to 22 (subject 7), but not all assessments were performed at each visit. All patients had at least one visit per year since ERT was initiated.

### Infusion details

All patients received elosulfase alfa at 2 mg/kg/week. Eight patients did not miss any planned infusions, with exception of two or three recent infusions due to the COVID-19 pandemic. Case 6 missed six infusions due to hypersensitivity reactions. Case 9 missed one or two infusions per month in a 1-year period after the last follow-up visit due to familial problems, but resumed treatment thereafter.

None of the patients, except case 6, had anaphylactic reactions to ERT. After the 16th weekly infusion, case 6 presented with vomiting, nausea, hypotension, urticaria on the trunk, and angioedema on the eyelids, which developed in approximately 90 min. A skin prick test was performed and confirmed a strong IgE-dependent allergic reaction. A desensitization protocol was initiated. During the first day of desensitization, hypotension occurred, and ERT was discontinued for 1 month on the parents’ request. Desensitization was resumed successfully after 1 month without further incidents. The patient is currently (after a 1-year protocol) being treated with 4-h infusions without any premedication.

### Diagnosis

Ages at diagnosis ranged from 2 weeks in case 1 to 16.1 years in case 8 (Table 1). Cases 1 and 2 were diagnosed with Morquio A syndrome before the onset of symptoms, due to sibling history (older sibling previously diagnosed with the disease). In the other patients, first symptoms of Morquio A syndrome observed by the parents were pectus carinatum (cases 3, 4 and 7), scoliosis (case 4), kyphoscoliosis (case 6), and growth retardation (cases 5, 8, 9, and 10). Patients were referred for diagnosis by a pediatrician (cases 3, 5, 7, 8, 9) or by an orthopedic surgeon (cases 4, 6, and 10). All patients had homozygous mutations in the *GALNS* gene (Table 1). All mutations were previously defined as likely pathogenic or pathogenic.

### Clinical findings before initiation of ERT

Table 2 summarizes clinical data collected during physical and clinical examinations before treatment initiation. All patients had at least some joint and skeletal abnormalities at the time ERT was started. Other frequent observations were recurrent respiratory infections (N = 10), corneal clouding (N = 9), coarse facial features (N = 9), hearing loss (N = 6), hepato(spleno)megaly (N = 6), and cardiac abnormalities (N = 6). It should be noted that some clinical manifestations may have been missed due to the young age of the patient (e.g. inability to perform a visual acuity test) or a test not being performed before or at treatment initiation. For case 7, physical examination data reported at 0.3 years after treatment initiation are included since no earlier data were available, as ERT was started in a different center.

**Table 2:** Clinical findings before or at initiation of enzyme replacement therapy^a^

Case	Corneal clouding	Hearing loss	Joint/skeletal abnormalities	Coarse face	Abdominal abnormalities	Cardiovascular disease	Other
1	Yes (mild)	No	Wrist laxity, shoulder stiffness, hip dysplasia, enlarged head, dysostosis multiplex, pectus carinatum	No	Hepatomegaly, umbilical hernia	No	Hypospadias, recurrent respiratory infections, CTS
2	Yes	Conductive type C	Wrist laxity, genu valgum, pectus carinatum, increased lumbar lordosis, mild kyphosis, dysostosis multiplex	Yes (mild)	No	No	Recurrent respiratory infections, tonsillar hypertrophy
3	No	No	Wrist laxity, genu valgum, hip dysplasia, pectus carinatum, minimal lumbar scoliosis, dysostosis multiplex	Yes (mild)	No	No	Recurrent respiratory infections, tonsillar hypertrophy
4	Yes (minimal)	Conductive	Wrist laxity, elbow stiffness, mild kyphosis, scoliosis, genu valgum, pectus carinatum, dysostosis multiplex	Yes (mild)	Hepatosplenomegaly	Mild mitral insufficiency	Recurrent pulmonary infections
5	Yes (mild)	NA	Mild wrist laxity, hip dysplasia, genu valgum, pectus carinatum, mild kyphosis, dysostosis multiplex	Yes (mild)	Hepatomegaly, umbilical hernia	Mitral valve thickening, mild mitral/aortic insufficiency	Recurrent respiratory infections, CTS, moderate upper airway obstruction due to adenoid and tonsillar hypertrophy, fecal incontinence
6	Yes (minimal)	Conductive type B & C and sensorineural	Wrist laxity, mild joint stiffness, genu valgum, pectus carinatum, mild kyphoscoliosis, dysostosis multiplex	Yes (mild)	Hepatomegaly, umbilical hernia	Mitral/aortic valve thickening, mild mitral insufficiency, respiratory sinus arrhythmia,	Recurrent respiratory infections, VA right eye 5/10, adenoid/tonsillar hypertrophy
7	Yes	No	Wrist laxity, joint stiffness, genu valgum, pectus carinatum, kyphosis, dysostosis multiplex	Yes (moderate)	No	No	Recurrent respiratory infections, VA 8/10 both eyes, papilledema, adenoid/tonsillar hypertrophy
8	Yes (mild)	Conductive type B & C	Wrist laxity, mild joint stiffness, genu valgum, hip dysplasia, ankle deformities, mild pectus carinatum, mild kyphosis, thoracolumbar scoliosis, dysostosis multiplex	Yes (Moderate)	Hepatomegaly	Mild aortic and mitral insufficiency	Recurrent respiratory infections, adenoid/tonsillar hypertrophy
9	Yes (mild)	Sensorineural	Wrist laxity, joint stiffness, pectus excavatum, kyphoscoliosis, genu valgum, bilateral patellar dislocation, dysostosis multiplex	Yes (Moderate-Severe)	Hepatosplenomegaly	Aortic insufficiency, mild aortic stenosis, mild mitral insufficiency	Recurrent respiratory infections, adenoid/tonsillar hypertrophy, muscle weakness
10	Yes (mild)	Sensorineural	Wrist laxity, mild joint stiffness, genu valgum, pectus excavatum, thoracolumbar scoliosis, dysostosis multiplex	Yes (mild)	Hepatomegaly	Mild mitral insufficiency	Recurrent respiratory infections, adenoid/tonsillar hypertrophy

^a^For case 7, physical examination data were first reported at 0.3 years after treatment initiation

CTS: carpal tunnel syndrome; NA: not available; VA: visual acuity

### Endurance

**Fig. 1:**
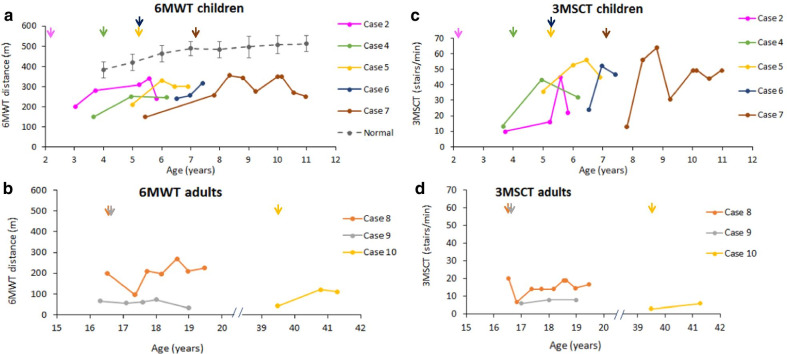
Six-minute walk test (6MWT) distance over time in children (**a**) and adults (**b**) and 3-min stair climb test (3MSCT) results over time in children (**c**) and adults (**d**). Arrows indicate ages at initiation of enzyme replacement therapy (ERT). In cases 4, 5, 7, 8, 9, and 10, the first 6MWT measurement was recorded at or before initiation of ERT; baseline 3MSCT data were available for cases 4, 5, 8, and 10. All other data were collected after initiation of ERT. The dashed line in figure A represents normal 6MWT distances by age for children 4–11 years old [13]. Error bars represent standard deviations

All reported 6MWT distances were considerably below normal values (i.e. below the mean 6MWT distance in healthy children at 4 years of age, which is 383 m) [13], but remained relatively stable or increased over time in most patients (Fig. 1a, b). 6MWT data at initiation of ERT (baseline) were available for three children (cases 4, 5 and 7) and all adults (cases 8–10). Cases 4, 5, 7, 8 and 10 showed increases in walking distance from baseline to last follow-up (case 4: from 150 to 245 m after 2.1 years; case 5: from 210 to 300 m after 1.7 years; case 7: from 150 to 250 m after 3.9 years; case 8: from 198 to 225 m after 3 years; case 10 from 42 to 110 m after 1.8 years). Case 9 had stable results in the first 2 years and a decline at 3 years (from 66 to 33 m after 3 years). This patient also required rest and support to perform the endurance tests. It should be noted that case 9 was diagnosed at 16.6 years old and he was not compliant to the weekly treatment (discontinued treatment many times).

3MSCT results fluctuated considerably over time in the children, but increased between the first and the last measurement in all of them (Fig. 1c, d); 3MSCT results of the adults were relatively stable. Baseline 3MSCT data were available for cases 4, 5, 8 and 10. All these patients showed at least some improvement from baseline to last follow-up, except case 8 who showed a minor decline.

Endurance data of cases 1 and 3 are not included in Fig. 1 as both patients had only one measurement, or two measurements with very short interval and no pre-ERT baseline. Results at last follow-up were 265 m in the 6MWT and 36.7 stairs/min in the 3MSCT for case 1 (at 3.7 years of age) and 125 m and 16 stairs/min, respectively, at 4.6 years of age for case 3.

### Anthropometrics

Height/length and weight data were available for all cases (Additional file 1). Data before treatment initiation were reported for all cases, except case 9.

Among the infants, who both started ERT around 2 years of age, case 1 had a length within 2 standard deviations of Centers for Disease Control and Prevention (CDC) reference curves at treatment initiation (z-score -0.97), while length was slightly below normal in case 2 (z-score −2.06). The older children had baseline height z-scores ranging between −2.56 and −4.97 (Additional file 1). Baseline weight z-scores were below normal (z-score < 2) in cases 5 and 6. All three adults (cases 8–10) had very short statures, with standing heights ranging from 99 to 103 cm (z-scores −8.5 to −9.7) and weights between 21 and 25 kg (z-scores −9.4 to −13.4). All patients > 2 years of age, except cases 7, 8 and 9, were overweight at baseline, which means that body mass index (BMI) was above the 85^th^ percentile of CDC norms in patients of 2–20 years of age or > 25 kg/m^2^ in older patients (Additional file 1).

**Fig. 2:**
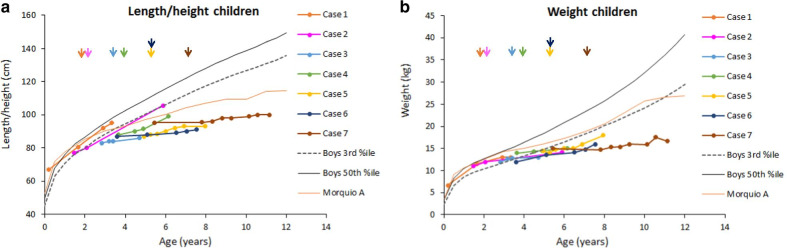
Stature and weight over time. Length (0 to 2 years) and height (2 to 12 years) for age curves (**a**) and weight for age curves (**b**) in infants and children. Arrows indicate age at initiation of enzyme replacement therapy. Dashed and full black lines represent 3rd and 50th percentiles for healthy boys (CDC https://www.cdc.gov/growthcharts/clinical_charts.htm), respectively; full orange lines represent means for untreated Morquio A syndrome (boys) [14]. Reference curves for girls are not included as these largely overlap with the curves for boys in this age range

All infants and children showed increases in length/height after initiation of ERT (Fig. 2). Growth curves of the two infants (cases 1 and 2) appeared to follow CDC reference curves after treatment initiation. Length/height z-scores slightly increased from −0.97 to −0.63 after 1.5 years of treatment in case 1 and from −2.06 to −1.8 after 3.7 years in case 2. In the children who started ERT after 3 years of age, height increasingly deviated from reference growth curves over time despite treatment, and continued to follow Morquio A population curves (Fig. 2) [14]. All infants and children with follow-up data, except case 1, had low weight (z-score < −2.0) at last follow-up. Follow-up data in adults (cases 8 and 9) showed no increases in height after initiation of ERT and slight increases in weight. BMI remained relatively unchanged in most patients, with exception of case 4, who showed a normalization (from the 94th to the 52th percentile), case 7, who showed a reduction from the 80th to the 29th percentile, and case 9, who changed from the 79th to 90th percentile, after 2 to 4 years of treatment (Additional file 1).

### Skeletal and joint abnormalities

All patients showed skeletal abnormalities before treatment initiation (Table 2). Frequent findings were wrist laxity (N = 10), pectus carinatum or excavatum (N = 10), genu valgum (knock knees; N = 9), kyphosis/scoliosis/kyphoscoliosis (N = 9), and joint stiffness (N = 7). Radiology examinations showed dysostosis multiplex in all patients. Although follow-up data were difficult to interpret, they did not suggest meaningful improvements in skeletal abnormalities over time. An exception was case 2, for whom an improvement of kyphosis was reported after 3.7 of 4.2 years of treatment. Follow-up data of the youngest patient (case 1) suggested progression of skeletal abnormalities. This patient showed mild joint stiffness and hyperlaxity and unilateral hip joint dysplasia at initiation of ERT at 1.8 years of age, but developed pectus carinatum, scoliosis and bilateral genu valgum within the next 1.5 years.

Joint range of motion was mainly reported for shoulders, elbows, knees and hips. Case 2 maintained normal joint range of motion over 3.7 years of ERT. All other cases showed joint motion limitations at baseline, including genu valgum (cases 3, 4, 5, 6, 7, 8, 9, 10), and stiffness (flexion, extension, or rotation limitations) of the shoulders (cases 1, 6, 8, 10), elbows (cases 3, 4, 7, 9, 10), knees (cases 8, 10), and/or hips (cases 4, 5, 7, 9, 10). There was no improvement or worsening in joint range of motion over time in any of these patients.

### Cardiorespiratory function

All echocardiographic investigations were performed by the same physician. Three children (cases 4, 5, and 6) and all three adults (cases 8–10) had mitral insufficiency before initiating ERT; cases 5, 6, 8, and 9 also had aortic insufficiency or valve thickening (Table 2). Cardiac valve disease resolved in case 6 after 1.8 years of treatment, and remained stable in cases 4 and 5. Case 8 showed mild aortic and mitral insufficiency when ERT was started and severe aortic insufficiency after 2.2 years of treatment. Case 9 had pulmonary hypertension, second-degree aortic insufficiency, mild aortic constriction and mild mitral insufficiency at baseline and at all follow-up examinations. Prophylactic penicillin use was recommended for infective endocarditis. In case 10, echocardiography revealed a diastolic relaxation of the left ventricle with slight mitral insufficiency and an ejection fraction of 72% at treatment initiation and second degree aortic insufficiency, and an ejection fraction of 70% after 2.5 years of treatment. Pulmonary hypertension resolved by last follow-up.

Four patients (cases 1, 2, 3 and 7) initially had normal cardiovascular function. Of those, one (case 7) developed mitral, tricuspid and aortic valve insufficiency after 2 years on treatment. Cases 1, 2, and 3 kept normal cardiovascular function after 0.8, 3.7, and 1.2 years of ERT, respectively.

Pulmonary function tests were performed at regular intervals after initiating ERT in five patients (cases 4, 7, 8, 9, and 10) and showed restrictive pulmonary disease in four of them (cases 4, 8, 9, and 10). Restrictive pulmonary disease worsened over time in case 9, but no progression was seen in the other patients.

### Ear, nose, throat

Hearing loss was reported for six patients (Table 2). Among these, four were diagnosed with conductive hearing loss (cases 2, 4, 6, and 8), mostly type B (likely due to otitis media) and/or type C (indicating Eustachian tube dysfunction). The two oldest patients (cases 9 and 10) and case 6 had sensorineural hearing loss. In addition, eight patients had upper airway obstruction due to adenoid and/or tonsillar hypertrophy (cases 2, 3, 5, 6, 7, 8, 9, and 10). Related to this, case 5 had ventilation tube insertions and adenoidectomy at 5.7 years of age, case 6 had three ventilation tube insertions and one adenoidectomy before 4 years of age, and case 8 had a tonsillectomy and an adenoidectomy at 16.5 years of age.

Hearing loss in case 8 and 10 remained stable. In case 8, otitis media was reported at 0.4 years after treatment initiation. Cases 6 and 10 started using hearing aids at 8 and 24 years of age, respectively. Follow-up ear/nose and throat investigations were planned, but have not yet been undertaken due to the COVID-19 pandemic.

### Vision

All patients except case 3 had corneal clouding at first measurement. Cases 6 and 7 also showed reduced visual acuity (Table 2). Five patients had both baseline and follow-up data (cases 1, 2, 6, 8 and 10). Corneal clouding remained present in cases 6, 8, and 10 after around 2 to 3 years of treatment. Cases 1 and 2 had mild corneal clouding before initiation of treatment, which resolved in case 1 after 0.8 years and in case 2 after 3.7 years of treatment. The degree of corneal clouding was subjectively graded by a single physician as mild (+), moderate (++), or severe (+++).

### Neurological and mental function

Neurological examination and/or spinal or brain magnetic resonance imaging (MRI) data were available for all patients except case 3, showing abnormalities in cases 1, 4, 5, 6, 7, 8, 9, and 10, and no abnormalities in case 2.

Cranial MRI in case 1 at 3.8 years of age revealed mild dilation of the third ventricle and perivascular spaces at the right frontal lobe. Cervical, thoracic, and lumbar MRI showed enlarged and biconcave vertebral bodies, but a normal spinal cord. In case 4, cranial and spine MRI at 4.6 years of age showed diffuse spinal cord compression on vertebral body and gibbus deformities in the lower thoracolumbar region and sacrococcygeal region, as well as craniocervical compression and myelomalacia. Case 5 had a cerebral MRI during a hospitalization for fecal incontinence shortly before initiatioin of ERT (at 5 years of age), showing spinal cord compression at C2 level but a normal cerebellum. An electromyogram (EMG) ruled out tethered cord syndrome. In case 6, spinal stenosis was reported at 2 years and spinal cord compression at 5 years of age (just before ERT was started), but without clinical symptoms. In case 7, spinal stenosis was reported at 8 years of age. In case 8, MRI results at 17 years of age showed spinal cord compression due to kyphosis at the upper thoracic region, and spinal cord compression and myelomalacia at the cranio-cervical region due to a narrow foramen magnum, causing hydrocephaly and some peripheral nervous system symptoms. At 18.6 years of age, a repeat spinal MRI in this patient showed worsening of spinal cord compression at the cranio-cervical region and hydrocephaly, requiring surgical decompression around 19 years. In case 9, a neurological examination at 17 years of age showed reduced muscle strength resulting in poor mobility. Deep tendon reflexes in this patient were minimal in the upper extremities and absent in the lower extremities. In case 10, MRI at 32 years of age showed spinal cord compression at the foramen magnum, upper cervical, lower thoracic and lumbar levels. Stenosis at the cranio-cervical junction resulted in myelomalacia. Spinal cord compression progressed over time; at last follow-up (40 year of age), an EMG showed patchy myogenic motor unit potentials, but otherwise no abnormalities.

Denver Developmental Screening Test II data were reported at 2.6 years of age for case 1, at 1.7 years of age for case 2, and at 4.5 years of age for case 3. These showed a delay in fine motor skills, but otherwise no abnormalities in case 1, a delay in language and gross motor skills in case 2, and no abnormalities in case 3. Intelligence quotient, as determined by the Stanford-Binet test in cases 4, 5 and 6, the WISC-R in cases 7, 8 and 9, and the WAIS in case 10, was within normal limits in all children and adults, except case 9, who had cognitive impairment (intelligence quotient [IQ] 52) at 17.2 years of age, which was not considered associated with the diagnosed Morquio A syndrome [15].

### Visceral involvement

Liver and spleen size were evaluated by physical examination and ultrasonography in all patients. Seven patients were reported to have hepatomegaly at treatment initiation (cases 1, 4, 5, 6, 8, 9 and 10) (Table 2). Case 9 and case 4 also had an enlarged spleen. Hepatomegaly resolved in cases 1, 4, 8, 9 and 10 at last follow-up, but persisted in cases 5 and 6.

Three patients (cases 1, 5, and 6) had an umbilical hernia at the time treatment was initiated. For case 5, an inguinal hernia repair was also reported shortly after initiation of ERT.

## Discussion

The present case series provides a better insight into the genotypic and phenotypic characteristics of a small group of Morquio A patients from Turkey, and provides real-life evidence on the long-term impact of ERT on the disease in clinical practice in a wide age range.

Mutational analysis and clinical data suggest that the majority of patients in the series had a classical (severe) Morquio A phenotype. Seven of the ten mutations reported in our case series have been described in previous publications [16–20]. Three of these i.e. p.P179S (case 1), p.W141R (case 7), and p.Q473X (case 9) were previously reported for Turkish patients [16–18], whereas p.C308R (case 3), p.L36P (case 4), p.R90W (case 5), and p.C308R (case 8) have been described in other countries [19, 20]. All these mutations have been associated with a classical phenotype, except L36P, which was linked to non-classical disease [16–20]. Our data support these genotype–phenotype correlations. In accordance with a classical Morquio A phenotype, cases 1 and 3 showed early onset of disease (before 2 years of age), including corneal clouding and skeletal dysplasia, and cases 5, 7, 8, and 9 showed short stature, poor mobility, skeletal dysplasia, corneal clouding, cardiac valve disease, and restrictive and obstructive respiratory disease. Case 4 showed less severe growth impairment, minimal corneal clouding, no ENT abnormalities, and minimal cardiac abnormalities, confirming a non-classical phenotype. To our knowledge, phenotypes associated with the mutations of case 2 (p.V141R), case 6 (p.R219X), and case 10 (p.E450K) have not been previously described in the literature. Based on their stature and clinical characteristics at initiation of ERT, cases 2, 6 and 10 can probably be classified as having a classical phenotype.

As could be expected based on the progressive natural history of Morquio A syndrome, the three adults in the case series showed more severe disability than the children. Endurance in the 6MWT and 3MSCT was most severely impaired in the adults, confirming findings from the Morquio A Clinical Assessment Program (MorCAP) natural history study, which show a decrease in these measures over time [21]. The adult patients also showed the greatest deviations from normative height and weight curves, confirming natural history data [2, 22]. In addition, they appeared to have more severe musculoskeletal, cardiorespiratory, and neurological manifestations than the younger patients. Corneal clouding, hearing impairment and skeletal and joint abnormalities (mainly kyphosis, pectus carinatum, and joint laxity) were also commonly observed in the younger patients, including the two infants, stressing the importance of these manifestations in the early diagnosis of Morquio A syndrome. Whereas most children had conductive hearing loss, sensorineural hearing loss was reported for two of three adults, suggesting development of conductive hearing loss (due to otitis media) in the early disease course, and mixed or sensorineural hearing loss later in life, as also reported for MPS II [23].

Overall, the longitudinal data after initiation of ERT suggest an impact of treatment on the progression of some clinical manifestations. The endurance outcomes appear to largely confirm published data. In line with the pivotal elosulfase alfa clinical trial [10], the adult patients showed a stabilization in endurance after initiation of treatment for up to 3 years. In the children, overall endurance also appeared to stabilize or improve over time. The most relevant findings in the infants were related to growth. Despite their classical phenotype, cases 1 and 2 showed slight increases in height z-scores after treatment initiation at 1.8 and 2.1 years of age and maintained a length/height within normal limits (z-scores -0.63 and -1.8) up to around 3 and 6 years of age, respectively. In older children, who initiated ERT between 3.4 and 7.1 years of age, height was already significantly below normal at baseline and further deviated from normal growth curves after treatment initiation.

Our growth data in the two infants are remarkable given the progressive deviation from normal growth curves after 2 years of age described for untreated patients with Morquio A syndrome [2, 14, 22]. These observations are particularly relevant considering the very limited data currently available for Morquio A patients starting ERT at this early age [22, 24, 25]. Our data confirm recent findings from a study of two siblings with Morquio A starting ERT at the ages of 54 months and 11 months. Over 4.5 years of treatment, growth of the youngest sibling deviated less from the norms than that of the older sibling, although a significant growth retardation was still seen after 2 years of age [24]. Other studies showed mixed results [22, 25]. A clinical study including 15 patients starting ERT at < 5 years of age also showed a trend towards improved growth versus untreated patients from the MorCAP study, although no increase in the mean z-score over time [22]. Another recent study including 12 patients starting ERT before 5 years of age, showed no effect of ERT on growth [25]. It is not clear why these studies have conflicting results. However, it should be noted that most patients that were included in both studies were older than 2 years when treatment was initiated. Together with our findings, the published data suggest that starting ERT very early in life can reduce, though not prevent, growth retardation in Morquio A patients. The earlier treatment is started, the better growth outcomes appear to be, although other factors such as genotype are also likely to have an impact. These findings support the international recommendation of starting treatment for Morquio IVA patients as early as possible [7].

The impact of (early) treatment initiation on other clinical manifestations was more difficult to determine. Apart from normal growth, case 2 also maintained normal joint range of motion and showed improvements in kyphosis and corneal clouding after 3.7 years of treatment. However, skeletal abnormalities continued to progress in the other cases after treatment initiation, although joint range of motion remained stable in all patients. Also, none of the other patients showed improvements in corneal clouding or vision, except case 1 in whom corneal clouding resolved after 0.8 years of ERT. The case series did not suggest a positive impact of ERT on cardiac function, with three patients showing a deterioration over time and the others showing no progression..

In conclusion, our case series provides real-life data on the natural history of Morquio A syndrome in patients from Turkey starting treatment with elosulfase alfa at different ages. Overall, our data appear to confirm clinical trial results showing a long-term stabilization of endurance after treatment initiation across ages [10]. In addition, growth data in the youngest patients suggest that very early intervention optimizes growth outcomes. This result provides support that a prompt diagnosis of this rare disease Morquio A, combined with the earliest initiation of ERT, would provide children the greatest potential improvement in health outcomes. Larger studies with long-term follow-up in infants are required to confirm these findings and to determine the long-term impact of early treatment initiation on other outcomes.

## Supplementary Information

**Additional file 1:** Length/height and weight z-scores at initiation of enzyme replacement therapy (ERT) and last follow-up. Description of data: Z-scores were calculated based on Centers for Disease Control and Prevention (CDC) growth charts (https://peditools.org/growthinfant/index.php for patients 0–2 years of age and https://peditools.org/growthpedi/index.php for older patients). Body mass index (BMI) percentiles were calculated using https://www.cdc.gov/healthyweight/bmi/calculator.html for patients 2–20 years and https://www.cdc.gov/healthyweight/assessing/bmi/adult_BMI/english_bmi_calculator/bmi_calculator.html for adults > 20 years.

## Data Availability

The datasets used and/or analyzed during the current study are available from the corresponding author on reasonable request.

## Abbreviations

3MSCT: 3-min stair climb test
6MWT: 6-min walk test
CDC: Centers for Disease Control and Prevention
EMG: Electromyogram
ENT: Ear-nose-throat
ERT: Enzyme replacement therapy
GAGs: Glycosaminoglycans
GALNS: N-acetylgalactosamine-6-sulfatase
IQ: Intelligence quotient
KS: Keratan sulfate
MorCAP: Morquio A Clinical Assessment Program
MPS: Mucopolysaccharidosis
MRI: Magnetic resonance imaging
SD: Standard deviation
uKS: Urinary keratan sulfate
WAIS: Wechsler Adult Intelligence Scale
WISC-R: Wechsler Intelligence Scale Revised test
