# Supplementary material for: Morquio A syndrome and effect of enzyme replacement therapy in different age groups of Turkish patients: a case series
Source: Orphanet J Rare Dis. 2021 Mar 22;16:144. doi: 10.1186/s13023-021-01761-0 (PMC7983100; doi:10.1186/s13023-021-01761-0)
Supplement: Supplementary file 1 — Additional file 1: Length/height and weight z-scores at initiation of enzyme replacement therapy (ERT) and last follow-up. Description of data: Z-scores were calculated based on Centers for Disease Control and Prevention (CDC) growth charts (https://peditools.org/growthinfant/index.php for patients 0–2 years of age and https://peditools.org/growthpedi/index.php for older patients). Body mass index (BMI) percentiles were calculated using https://www.cdc.gov/healthyweight/bmi/calculator.html for patients 2–20 years and https://www.cdc.gov/healthyweight/assessing/bmi/adult_BMI/english_bmi_calculator/bmi_calculator.html for adults > 20 years. [file 13023_2021_1761_MOESM1_ESM.docx]

**Additional file 1. Length/height and weight z-scores at initiation of enzyme replacement therapy (ERT) and last follow-up**

Z-scores were calculated based on Centers for Disease Control and Prevention (CDC) growth charts (<https://peditools.org/growthinfant/index.php> for patients 0-2 years of age and <https://peditools.org/growthpedi/index.php> for older patients). Body mass index (BMI) percentiles were calculated using <https://www.cdc.gov/healthyweight/bmi/calculator.html> for patients 2-20 years and <https://www.cdc.gov/healthyweight/assessing/bmi/adult_BMI/english_bmi_calculator/bmi_calculator.html> for adults >20 years.

| Case | Treatment initiation or first measurement^a^ | | | Last follow-up | | | |
| --- | --- | --- | --- | --- | --- | --- | --- |
|  | Height  z-score | Weight  z-score | BMI  %ile^b^ | Height z-score | Weight z-score | BMI %ile^b^ | Time on ERT (years) |
| 1 | -0.97 | -0.42 |  | -0.63 | -1.28 |  | 1.50 |
| 2 | -2.06 | -0.61 |  | -1.80 | -3.27 |  | 3.74 |
| 3 | -3.22 | -1.14 | 94 | -4.65 | -2.47 | 92 | 1.30 |
| 4 | -2.56 | -0.62 | 94 | -3.56 | -2.64 | 52 | 2.08 |
| 5 | -4.97 | -2.36 | 94 | -6.95 | -2.50 | 95 | 2.77 |
| 6 | -3.16 | -2.39 | 92 | -6.51 | -3.47 | 94 | 2.38 |
| 7 | -3.38 | -1.85 | 80 | -6.34 | -5.73 | 29 | 4.04 |
| 8 | -9.32 | -13.43 | 41 | -9.20 | -13.3 | 41 | 1.26 |
| 9 | -8.46 | -9.37 | 79 | -9.67 | -9.22 | 90 | 1.95 |
| 10^c^ | -9.70 | -10.29 | Overweight^d^ | NA | NA | NA | NA |

^a^For case 9, anthropometric data were first reported at 0.5 years after treatment initiation

^b^BMI was only calculated above 2 years of age

^c^Z-score calculated for 20 year old

^d^BMI >25 kg/m^2^

NA: not available
